# MINDFULNESS-BASED STRESS REDUCTION (MBSR): TREATMENT FOR COGNITIVE IMPAIRMENT POST CHEMOTHERAPY (CT)

**DOI:** 10.1093/geroni/igad104.1207

**Published:** 2023-12-21

**Authors:** Cecile Lengacher, Richie Reich

**Affiliations:** University of South Florida, Tampa, Florida, United States; University of South Florida, Tampa, Florida, United States

## Abstract

The purpose of this R01 trial was to evaluate the efficacy of MBSR(BC) compared to the Breast Cancer Education Support program(BCES) or Usual Care(UC) regimens for improvement in cognitive functioning among breast cancer survivors(BCS) who received (CT), or CT and radiation.

Methods: BCS (212) were randomized to MBSR(BC) (n=91) or BCES(90) or UC (n = 31) with MBSR(BC) and BCES participants attending a 6-week program. Measures of neuropsychological and perceived cognitive performance, psychological and physical symptoms were collected at baseline, 6, 12, and 26 weeks. Linear mixed models assessed the MBSR(BC) effects over time. Characteristics at baseline were tested as moderators of MBSR(BC) program effects.

Results: 212 BCS had a mean age of 57, 73% were White, non-Hispanic, 78% received CT and radiation; and 22% CT alone. All BCS improved across the study on all measures. For most measures, cognitive and symptom improvement effects followed the hypothesized pattern favoring MBSR(BC), followed by BCES and UC groups. Effect sizes were generally small, particularly for measures of cognition. The largest week 26 effect sizes were observed in the physical symptom fatigue, where the MBSR(BC) group improved with a 0.45 Cohen’s d effect over BCES and 0.65 over UC. Type of CT, radiation treatment, time since treatment, and informal and formal practice techniques of MBSR(BC) moderated the effects of MBSR(BC).

Conclusions: Although effects were modest, BCS receiving the MBSR(BC) and BCES groups improved in objective and subjective cognitive functioning and symptoms compared to UC. Longer follow-up studies are needed to determine sustained effects.

